# Supplementary material for: A general interfacial-energetics-tuning strategy for enhanced artificial photosynthesis
Source: Nat Commun. 2022 Dec 16;13:7783. doi: 10.1038/s41467-022-35502-z (PMC9758122; doi:10.1038/s41467-022-35502-z)
Supplement: Supplementary file 1 — Supplementary Information [file 41467_2022_35502_MOESM1_ESM.pdf]

Supplementary Information

A general interfacial-energetics-tuning strategy for enhanced artificial photosynthesis

Tian Liu,[1,2] Zhenhua Pan,[3]* Kosaku Kato,[4] Junie Jhon M. Vequizo,[5] Rito Yanagi,[6,7] Xiaoshan Zheng,[1] Weilai Yu,[8] Akira Yamakata,[4] Baoliang Chen[1], Shu Hu,[6,7] Kenji Katayama,[3] Chiheng Chu[1]*

[1] Faculty of Agriculture, Life, and Environmental Sciences, Zhejiang University, Hangzhou 310058, China

[2] Suzhou Institute for Advanced Research, University of Science and Technology of China, Suzhou 215000, China

[3] Department of Applied Chemistry, Faculty of Science and Technology, Chuo University, 1-13-27 Kasuga, Bunkyo, Tokyo 112-8551, Japan

[4] Faculty of Natural Science and Technology, Okayama University, 3-1-1, Tsushima-naka, Kita-ku, Okayama, Japan

[5] Research Initiative for Supra-Materials, Shinshu University, 4-17-1 Wakasato, Nagano-shi, Nagano 380-8553, Japan

[6] Department of Chemical and Environmental Engineering, Yale University, New Haven, Connecticut 06511, United States

[7] Energy Sciences Institute, Yale University, West Haven, Connecticut 06516, United States.

[8] Department of Chemical Engineering, Stanford University, Stanford CA 94305, United States

* Corresponding Author: zhenhua.20y@g.chuo-u.ac.jp, chuchiheng@zju.edu.cn

Chemicals

All chemicals were used as received without further purification. Acetic acid (> 99.7%), hydrogen peroxide, sodium phosphate dibasic, sodium dihydrogen phosphate, methanol (HPLC grade, >99.9%), formaldehyde (37%, w/w), resorufin sodium salt, amplex red, peroxidase (from horseradish), hydrogen peroxide solution (>30%, w/w), cobalt (II) nitrate, sodium tetrachloropalladionate, ammonium metavanadate, bismuth nitrate and *N*-(3-Dimethylaminopropyl)-*N'*-ethylcarbodiimide hydrochloride were obtained from Sigma Aldrich. Titanium oxide (>99.8%, 40 nm, rutile) was obtained from Aladdin. Carbon nitride (C_3N_4, thickness 1-5 nm) was obtained from XFNANO. All solutions were prepared using ultrapure water (>18.2 MΩ•cm) produced by Millipore Milli-Q Water Purification System.

Table S1 Comparison of H_2O_2 photosynthesis performance.

Photocatalyst	Experimental Conditions		Temperature	Rate (μM h^{-1})	AQY % (420 nm)	Electron Donor	AQY % (Full spectrum)	STH (%)	Ref.
	Gas	Light							
**Inorganic Photocatalyst**									
CoO_x/Mo:$BiVO_4$/(Ag/Pd)	O_2	AM 1.5	285K	9700 (irradiation area 4.5 cm^2)	13.1	H_2O	3.0	0.73	This work
CoO_x/Mo:$BiVO_4$/Pd	O_2	AM 1.5	285K	1425 (irradiation area 1.8 cm^2)	5.8	H_2O	1.2	0.29	1
Pd/TiO_2	Air	AM 1.5	Room temperature	150	-	H_2O	-	-	2
rGO/TiO_2/CoPi	O_2	λ > 320 nm	Room temperature	60	-	H_2O	-	-	3
GO	Air	Simulated sunlight	298K	50	-	H_2O	-	-	4
rGO/TiO_2/P	O_2	λ > 320 nm	Room temperature	30	-	H_2O	-	-	3
Au/$BiVO_4$	O_2	λ > 420 nm	298K	12	0.24	H_2O	-	-	5
$BiVO_4$	O_2	λ > 420 nm	298K	< 0.5	-	H_2O	-	-	5
**Organic Photocatalyst**									
RF/P3HT	O_2	λ > 300 nm	333K	3000	11	H_2O	-	1.5	6
COF-TfpBpy	O_2	420-700 nm	333K	2000	13.6	H_2O	-	1.08	7
RF523	O_2	420-700 nm	333K	2067	8	H_2O	-	0.5	8
Sb-SAPC	O_2	AM1.5	298K	588	17.6	H_2O	-	0.61	9
Co_1/AQ/C_3N_4	O_2	AM 1.5	Room temperature	62	-	H_2O	0.054	0.014	10
g-C_3N_4/BDI50	O_2	420-500 nm	298K	27.8	2.6	H_2O	-	0.13	11
g-C_3N_4/PDI/$RGO_{0.05}$	O_2	AM 1.5	298K	24	6.1	H_2O	-	0.2	12
g-C_3N_4	O_2	λ > 420 nm	Room temperature	175	4.3	H_2O	-	0.26	13
ZnPPc/NBCN	O_2	400-800 nm	Room temperature	11.4	-	H_2O	-	-	14
MRFS-7	O_2	AM 1.5	288K	284.25	-	H_2O	-	1.1	15
NH_2-UIO66(Zr)@OPA	O_2	λ > 420 nm	Room temperature	9700	-	71 vol% Benzyl Alcohol	-	-	16
cyano-C_3N_4	O_2	Simulated sunlight	Room temperature	16050	9.58	10 vol% Methanol	-	-	17

Table S2. Parameters for COMSOL Multiphysics simulation

Parameters	Values
Electron Affinity, χ_e	4.46 V
Energy band gap, E_g	2.4 eV
Effective density of states for conduction energy bands, N_C[18]	2.3×10^{19} cm^{-3}
Effective density of states for valence energy bands, N_V[18]	1.8×10^{19} cm^{-3}
n-type doping density, N_d[19]	3 *10^{17} cm^{-3}
vacuum permittivity e_0	8.85×10^{-12} F m^{-1}
Relative permittivity, e_r [19]	64
Electron and hole mobility μ_n, μ_p [20,21]	0.04 cm^2 V^{-1} s^{-1}
Hole lifetime[20,21]	40 ns
Majority carrier (electron) collection velocity (v_c)[19]	100 cm s^{-1}
Surface recombination velocity for hole at 110	0.1 m s^{-1}
Surface recombination velocity for hole at 010	10^{-5} m s^{-1}
Surface recombination velocity for electron at 110	0 m s^{-1}
Surface recombination velocity for electron at 010	10^{-5} m s^{-1}
CoO_x site work function	5.68 V

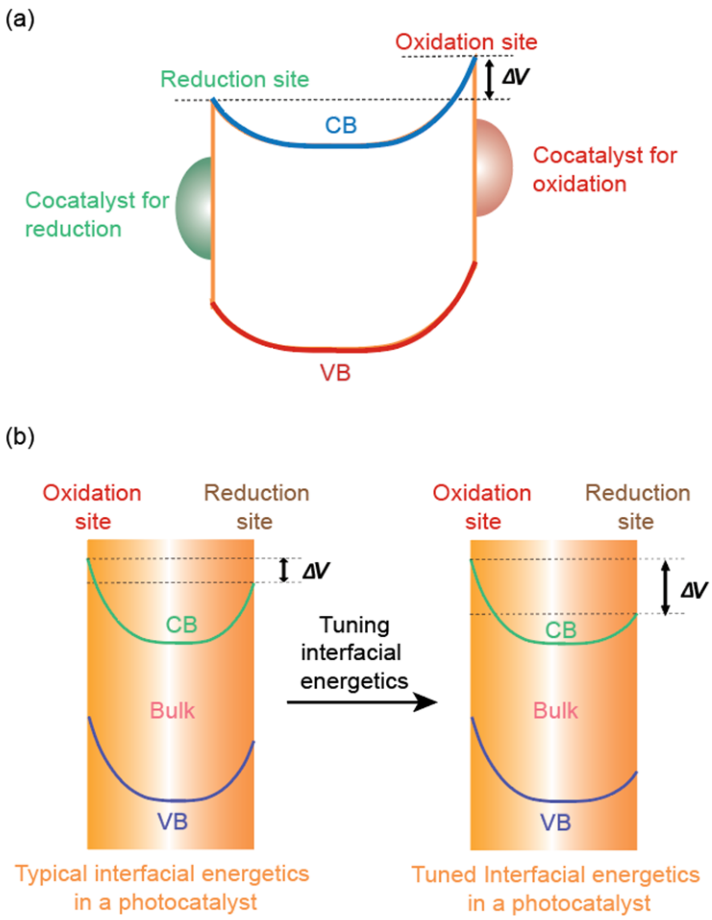

Figure S1. (a) Asymmetric energetics in a photocatalyst. (b) Tuning interfacial energetics in a photocatalyst. ΔV indicates the band offset potential between the reduction and oxidation sites.

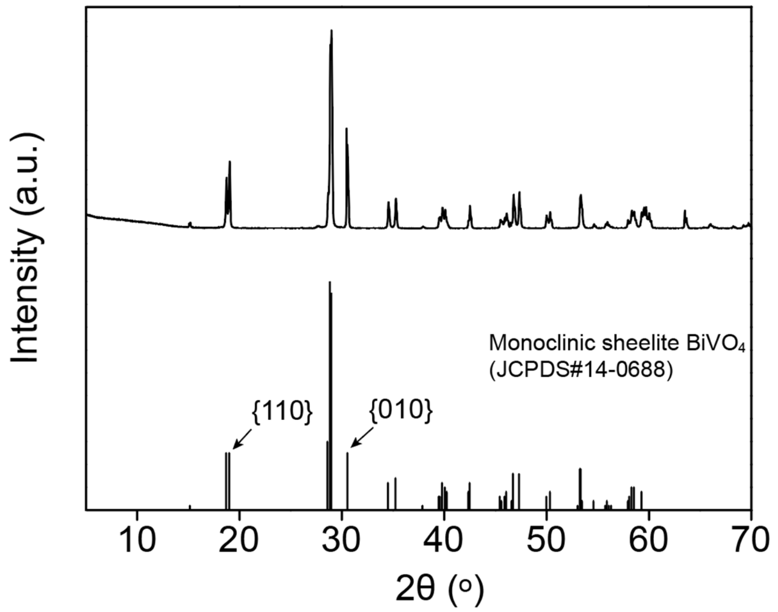

Figure S2. The XRD pattern of $BiVO_4$. The XRD pattern of $BiVO_4$ was in good agreement with the JCPDS standard card #14-0688, corresponding to monoclinic scheelite $BiVO_4$.

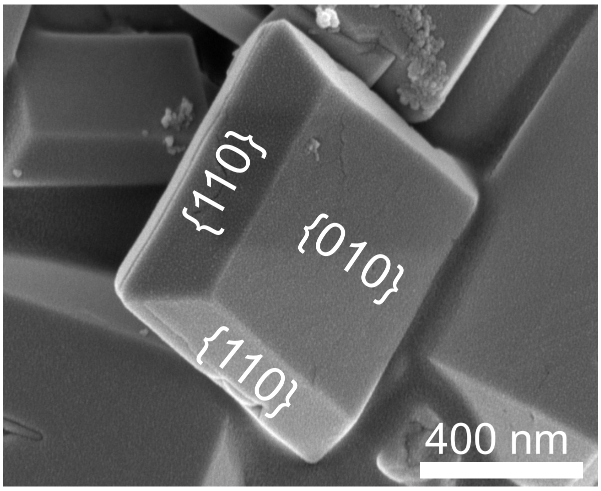

Figure S3. SEM image of $BiVO_4$ particles.

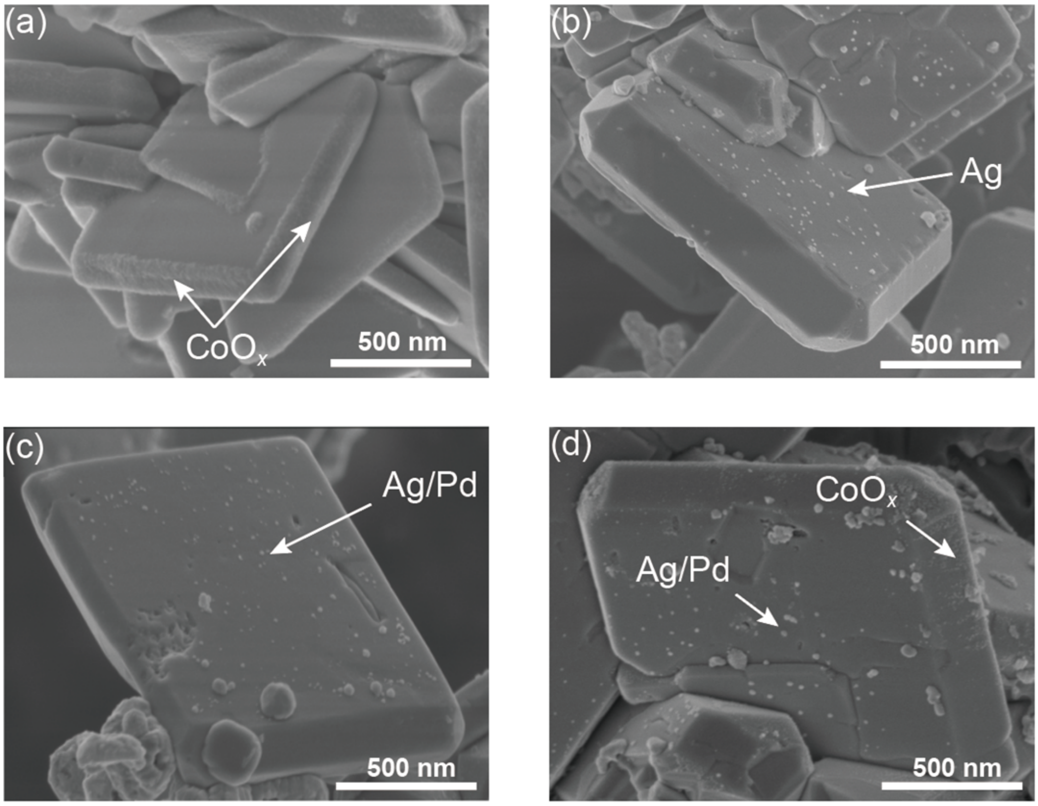

Figure S4. SEM images of (a) $CoO_x/BiVO_4$, (b) $BiVO_4/Ag$, (c) $BiVO_4/Ag/Pd$ and (d) $CoO_x/BiVO_4/(Ag/Pd)$.

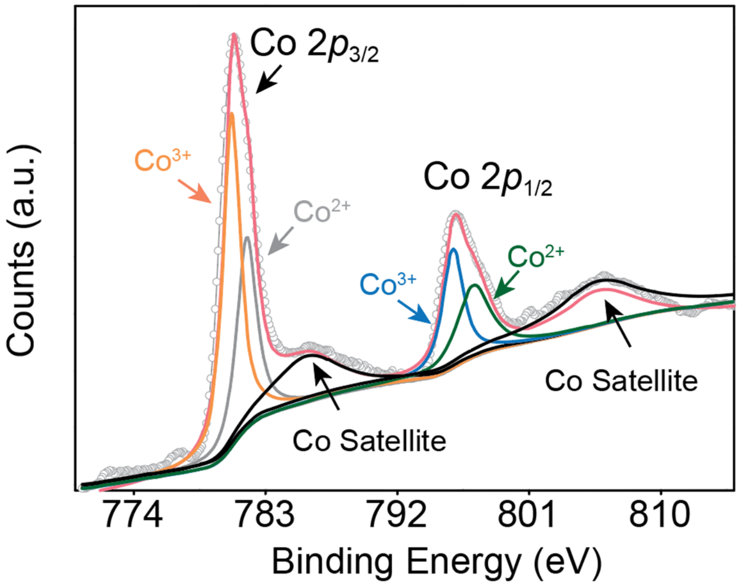

Figure S5. Co 2p XPS spectra of $CoO_x/BiVO_4$. The Co $2p_{3/2}$ peak can be deconvoluted to a Co^{2+} peak at 781.6 eV and a Co^{3+} peak at 780.6 eV. The area of Co $2p_{3/2}$ peak is twice of that of Co $2p_{1/2}$ peak and the binding energies of Co^{3+} and Co^{2+} are 780.6 and 781.6 eV, respectively. The other two peaks are the Co satellite peaks.

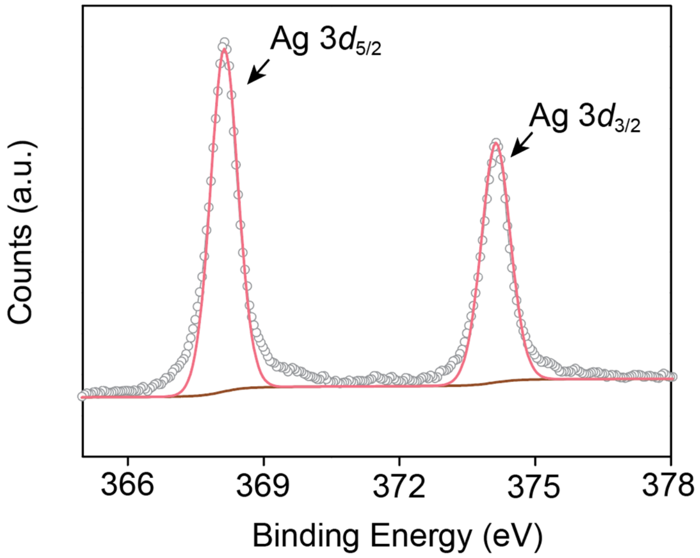

Figure S6. Ag 3d XPS spectra of $CoO_x/BiVO_4/Ag$. The binding energy of Ag $3d_{5/2}$ and $3d_{3/2}$ peaks were located at 368.1 eV and 374.2 eV, respectively.

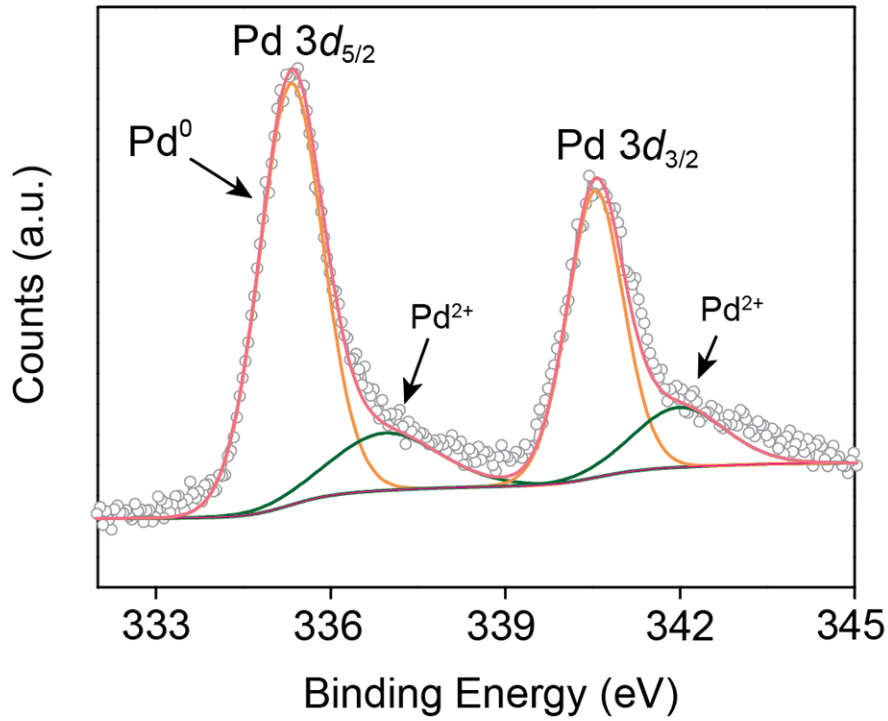

Figure S7. Pd 3d XPS spectra of $CoO_x/BiVO_4$/(Ag/Pd). The binding energy Pd $3d_{5/2}$ and $3d_{3/2}$ peaks were located at 335.1 eV and 340.6 eV, respectively. The binding energies of Pd^0 and Pd^{2+} are 335.1 and 337.0 eV, respectively.

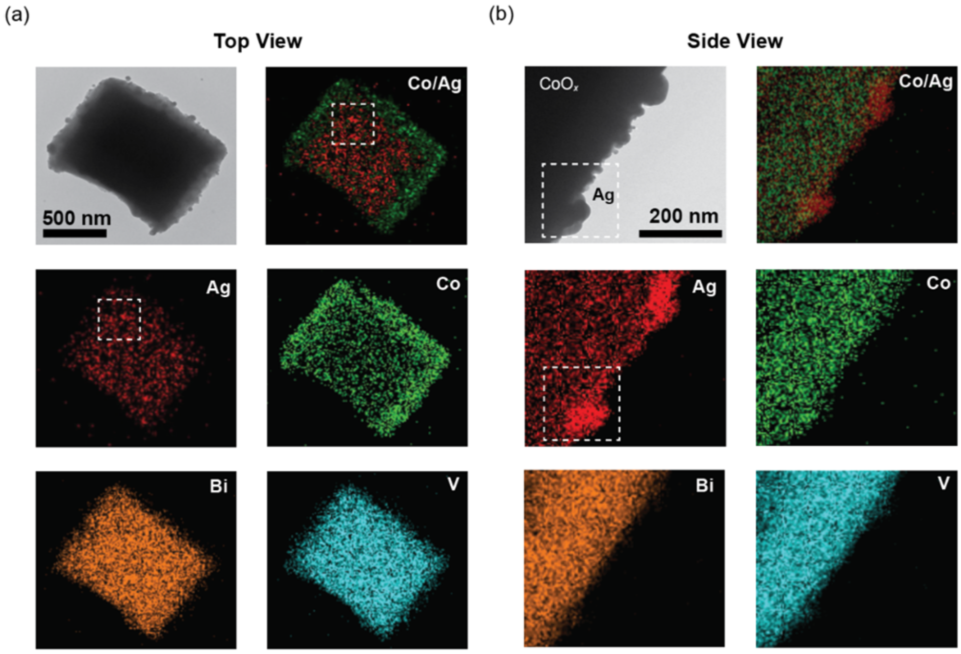

Figure S8. Top view (a) and side view (b) of STEM-EDS elemental mapping of Co and Ag particles loaded on $BiVO_4$.

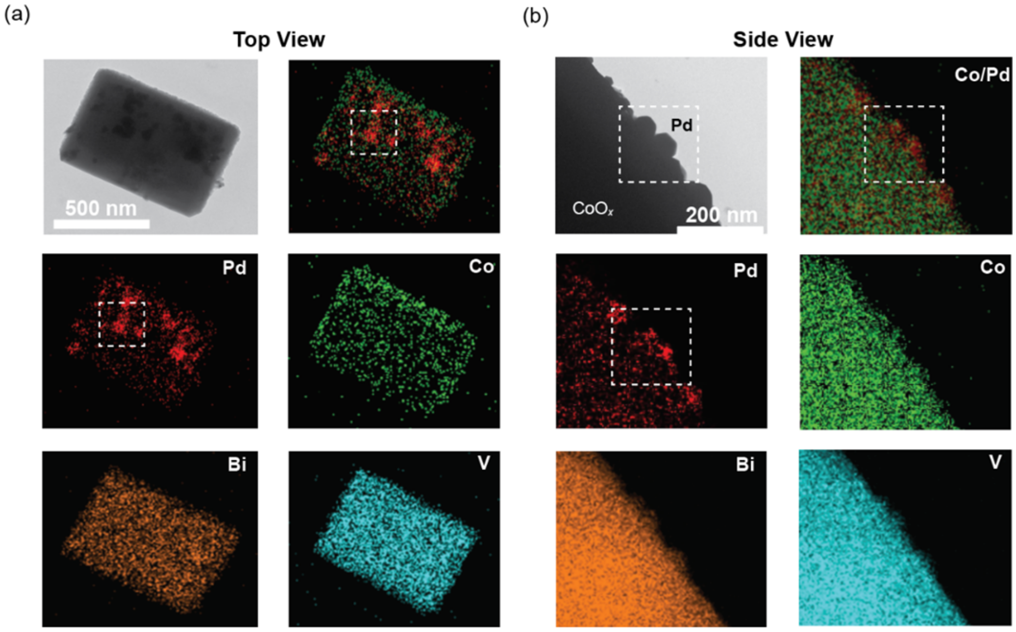

Figure S9. Top view (a) and side view (b) of STEM-EDS elemental mapping of Co and Pd particles loaded on $BiVO_4$.

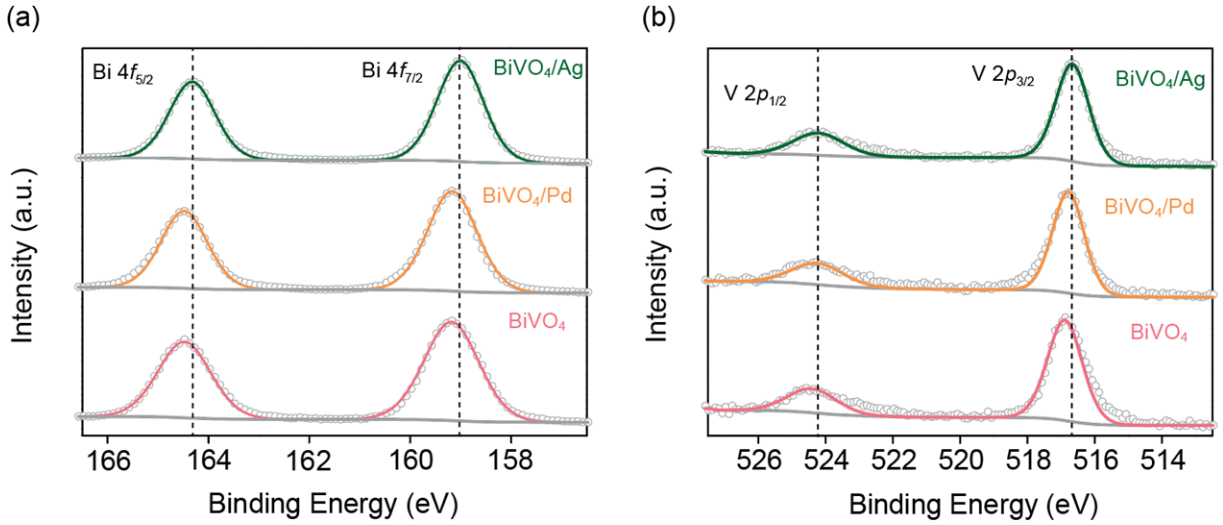

Figure S10. (a) Bi and (b) V XPS spectra of $BiVO_4$, $BiVO_4$/Pd and $BiVO_4$/Ag.

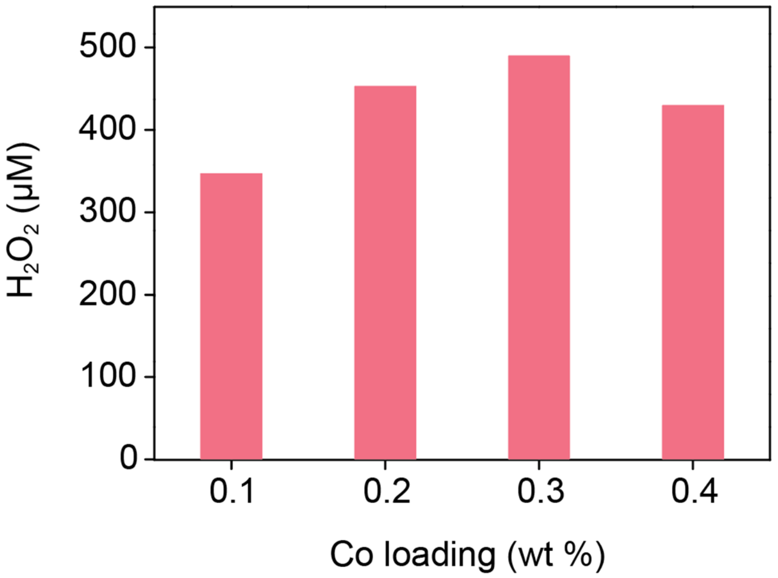

Figure S11. H_2O_2 generation over CoO_x/$BiVO_4$/(Ag/Pd) as a function of Co loading amount. Reaction conditions: photocatalyst amount, 1 mg ml^{-1} reactant solution, 50 ml pure water saturated with O_2; light source, light emitting diode (LED), 100 mW cm^{-2}, λ > 420 nm.

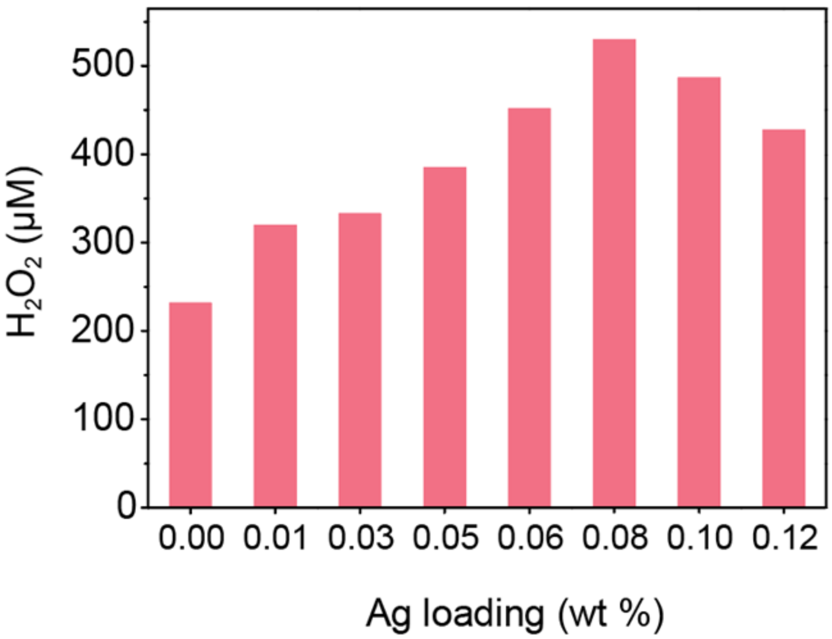

Figure S12. H_2O_2 generation over CoO_x/$BiVO_4$/(Ag/Pd) as a function of Ag loading amount. Reaction conditions: photocatalyst amount, 1 mg ml^{-1} reactant solution, 50 ml pure water saturated with O_2; light source, light emitting diode (LED), 100 mW cm^{-2}, $\lambda > 420$ nm.

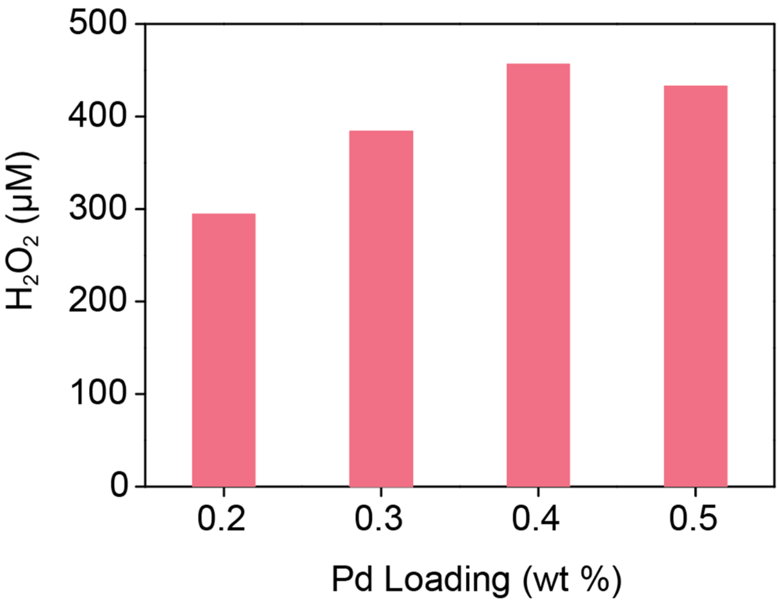

Figure S13. H_2O_2 generation over CoO_x/$BiVO_4$/(Ag/Pd) as a function of Pd loading amount. Reaction conditions: photocatalyst amount, 1 mg ml^{-1} reactant solution, 50 ml pure water saturated with O_2; light source, light emitting diode (LED), 100 mW cm^{-2}, $\lambda > 420$ nm.

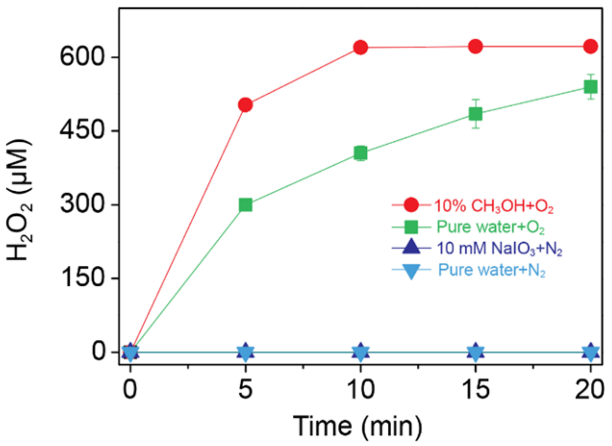

Figure S14. Time courses of photocatalytic H_2O_2 generation in 10%(v/v) CH_3OH, DI water, 10 mM $NaIO_3$ solution under N_2 condition, and DI water under N_2 condition. Reaction conditions: photocatalyst, 1 mg/mL; 50 ml reaction solution; light source, LED visible light, 300 mW cm^{-2}, $\lambda >$ 400 nm.

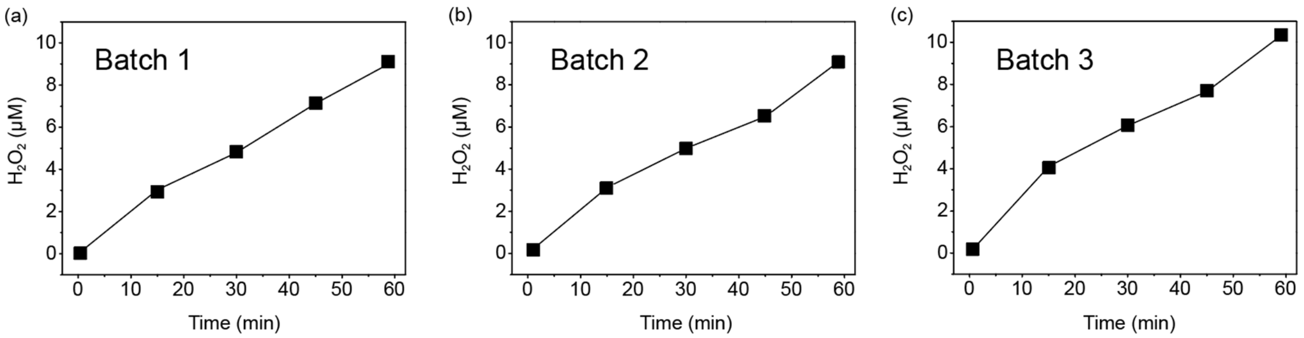

Figure S15. (a)-(c) Photocatalytic H_2O_2 generation from 3 individually synthesized batches of $CoO_x/BiVO_4/(Ag/Pd)$. Reaction conditions: photocatalyst amount, 1 mg ml^{-1}; reactant solution, 10 ml $ScCl_3$ water suspension (0.75 mmol $ScCl_3$, pH 6.9) with 100 mM H_3BO_3 saturated with O_2; light source, Xenon lamp, 100 mW cm^{-2}.

Section S1. Calculating the photogenerated charge-carrier distribution

The photon flux $\Phi(\lambda)$ from the lamp is shown in Fig. S17a. The reactor except for the window was wrapped by aluminum foils to preserve the light from escaping from the reactor. Therefore, all the incident light with wavelength < 520 nm (the light absorption edge wavelength of $BiVO_4$) is supposed to be absorbed by the $BiVO_4$ particles. As the particles were suspended in solution with stirring, individual particle was supposed to get irradiated homogenously from all direction. The window of the reactor for light illumination has an area of 7 cm^2. Therefore, the total number of incident photons into the reactor is 7 $cm^2 \times \Phi(\lambda)$. Based on Brunner−Emmet−Teller (BET) analysis, $BiVO_4$ particles exhibit a surface area of 1.5 m^2/g. During photocatalytic H_2O_2 generation, the suspension contained 0.05 g of particles, which gives a total surface area of 750 cm^2. Therefore, the photon flux on the surface of a particle, $\Phi_0(\lambda)$, is [7 $cm^2 \times \Phi(\lambda)$]/750 cm^2, as shown in Fig. S17b. The $\Phi_0(\lambda)$ in the range of $\lambda < 520$ nm which can be absorbed by $BiVO_4$ is integrated and gives a value of 1.7×10^{-3} μmol s^{-1} cm^{-2}. Based on the morphology of the $BiVO_4$ particle used in our simulation (Fig. S16), the surface area of each particle is estimated to be ~ 6.3 $\times10^{-8}$ cm^2. Therefore, the total photon flux absorbed by each particle ($G \times V$) is 6.5×10^{7} [#/s]. By dividing this value with the total volume of each particle, 6×10^{-13} cm^3, the generation rate per volume (G) with a value of 1×10^{20} [#/s cm^3] is obtained.

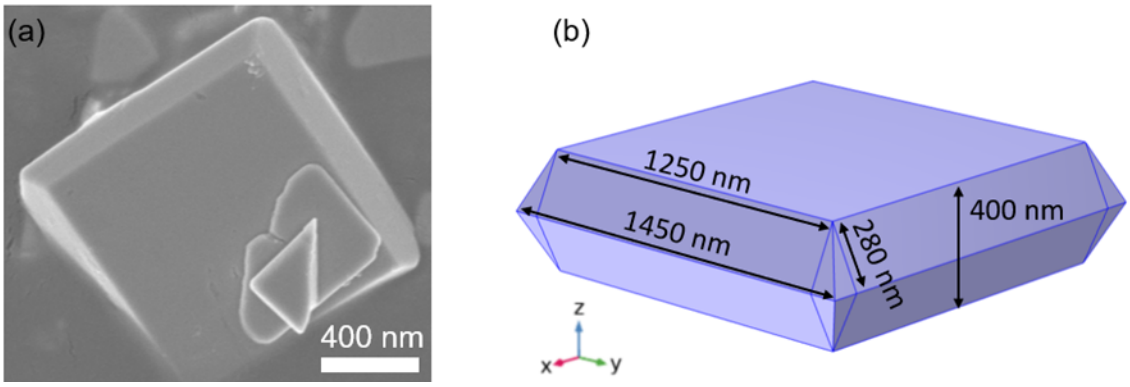

Figure S16. (a) SEM image of a $BiVO_4$ particle. (b) Morphology of the $BiVO_4$ particle simulated in COMSOL Multiphysics.

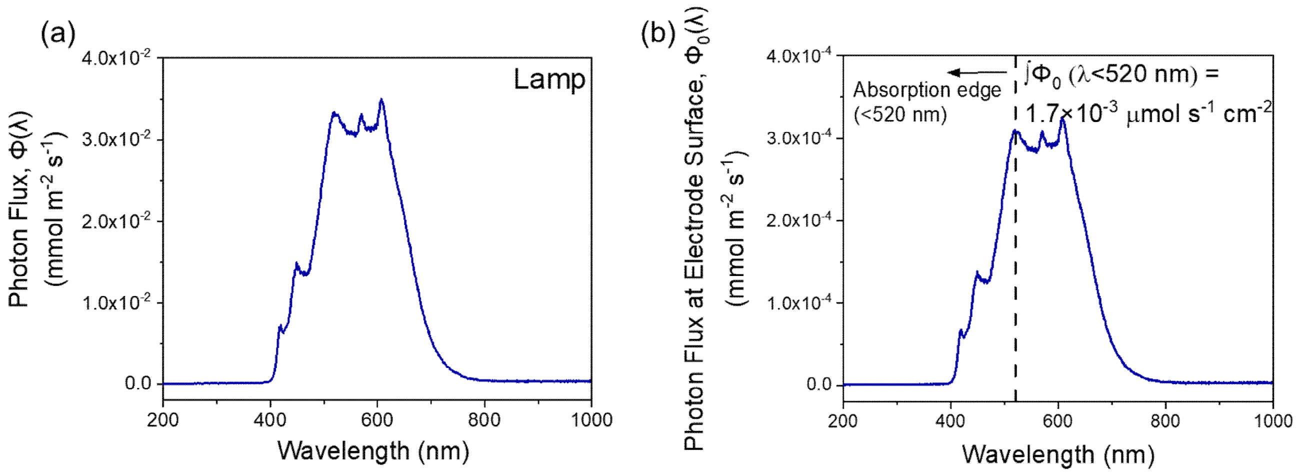

Figure S17. Photon flux $\Phi(\lambda)$ from the lamp (a) and (b) photon flux $\Phi_0(\lambda)$ on the surface of a $BiVO_4$ particle.

The photocarrier generation $G \times V$ is split into 6 directions as shown in Fig. S18. Given that the light irradiation is uniformly distributed on the particle surface, the generation $G \times V$ for each facet is set to be proportional to their surface areas. As a result, generation $G \times V$ for top/bottom and side facets are $1/4 \times G \times V$ and $1/8 \times G \times V$, respectively. Here, we calculate the generation rate $G(z)_{top}$ dependent on the penetration depth using the *z* direction as an example (Equations S1-S3). Note that $G(z)_{top}$ here is the summation of wavelength < 520 nm and only depends on the penetration depth. In addition, $G(z)_{top}$ only varies along the *z* axis and is constant along *x* and *y* direction.

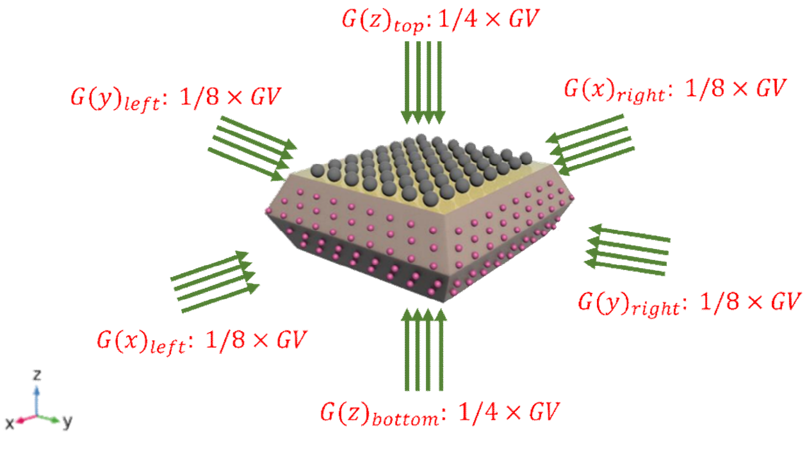

Figure S18. Generation rate for each facet.

$$G(z,\lambda)_{top} = \alpha(\lambda) * N_0(\lambda)e^{-\alpha(\lambda)z} \quad (1)$$

$$G(z)_{top} = \sum G(z,\lambda) \quad (2)$$

$$G(z)_{top} = \alpha * N_0 e^{-\alpha z} \quad (3)$$

Equation S1 shows the commonly used generation rate dependent on both penetration depth and wavelength. $\alpha(\lambda)$ is absorption coefficient and $N_0(\lambda)$ is the photon flux on photoabsorber surface, at each wavelength. Equation S2 shows the $G(z)_{top}$ in this study which only depends on penetration depth. α in Equation S3 is the wavelength-independent absorption coefficient and α^{-1} was defined to be 200 nm in our study. N_0 is a fitted value so that it satisfies Equation S4.

$$\int G(z)_{top} dV = \iiint G(z)_{top} dxdydz$$

$$= \iiint [-N_0 e^{-\alpha \times z}]\, dzdxdy = \iint (-N_0 e^{-\alpha \times z} + N_0)\, dxdy$$

$$= (-N_0 e^{-\alpha \times z} + N_0) \times top\ facet\ surface\ area$$

$$= 1/4 \times G \times V = 1.63 \times 10^7 [\tfrac{\#}{s}] \quad (4)$$

$G(z)$ can be obtained by adding $G(z)_{top}$ and $G(z)_{bottom}$ together. $G(x)$ and $G(y)$ can be obtained in a similar approach. By summing $G(x)$, $G(y)$, and $G(z)$, the generation rate on the whole particle, $G(x,y,z)$, is obtained. The obtained $G(x,y,z)$ is illustrated in Fig. S19.

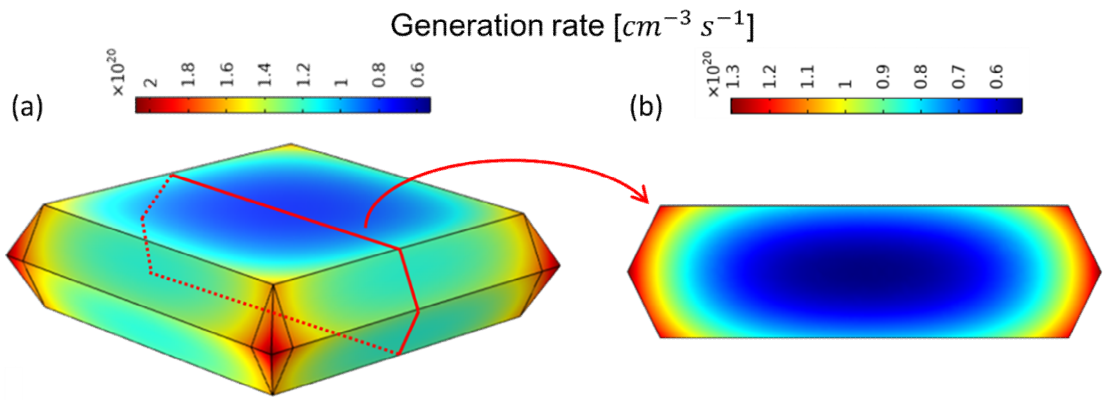

Figure S19. (a) Generation rate, $G(x,y,z)$, of a $BiVO_4$ particle and (b) that of a cross-sectional surface.

Section S2. Converting photocatalytic H_2O_2 generation rates to photocurrent densities

Because H_2O_2 may decompose as its concentration accumulated, the photocatalytic H_2O_2 generation rates in the first 5 min of $CoO_x/BiVO_4/Pd$ and $CoO_x/BiVO_4/(Ag/Pd)$ was applied for simulation. The H_2O_2 generation rates as shown in Fig. 2a were converted to photocurrent densities at the reduction and oxidation facets for the convenience of discussion.

The H_2O_2 generation rate of $CoO_x/BiVO_4/(Ag/Pd)$ is 3600 μM h^{-1} in 50 mL suspension, corresponding to a photocurrent of 9.63 mA. The suspension contains 0.05 g particles with a total surface area of 750 cm^2. Based on the SEM image of $BiVO_4$ particles (Fig. S16a), the sum of the areas of top and bottom facets is close to that of all side facets. To this end, the effective area for oxidation or reduction is ~ 375 cm^2. The photocurrent density for surface reactions is 9.63 mA/375 cm^2, i.e., 0.0257 mA cm^{-2}. The H_2O_2 generation rate of $CoO_x/BiVO_4/Pd$ is 1140 μM h^{-1}. By the same approach, its photocurrent density for surface reactions is calculated to be 0.0081 mA cm^{-2}.

Section S3. Simulation model

Following a previous study,[22] we regard a cocatalyst-loaded $BiVO_4$ particle as the combination of a solar cell for providing driving force and an electrocatalyst cell for catalyzing reactions as show in Fig. S20. The solar cell was simulated by COMSOL that gave its *J-V* curve under illumination. The electrocatalyst cell is composed of a cathode with Pd for H_2O_2 generation and an anode with CoO_x for O_2 evolution. These two half cells are individually investigated in three-electrode systems as shown in Fig. S21a. The *J-V* curve for O_2 evolution on the anode with CoO_x is fit with a Butler-Volmer equation to subtract the current for Co^{2+} oxidation. The *J-V* curves of the two half cells are combined to obtain a *J-V* curve for the two-electrode electrocatalyst cell as shown in Fig. S21b. The *J-V* curves of the solar cell and the electrocatalyst cell are drawn in the same coordination plan and their intersect corresponds to the photocurrent density of cocatalyst-loaded $BiVO_4$, as shown in Fig. S22. This photocurrent density is compared with the values from experimental H_2O_2 generation rates.

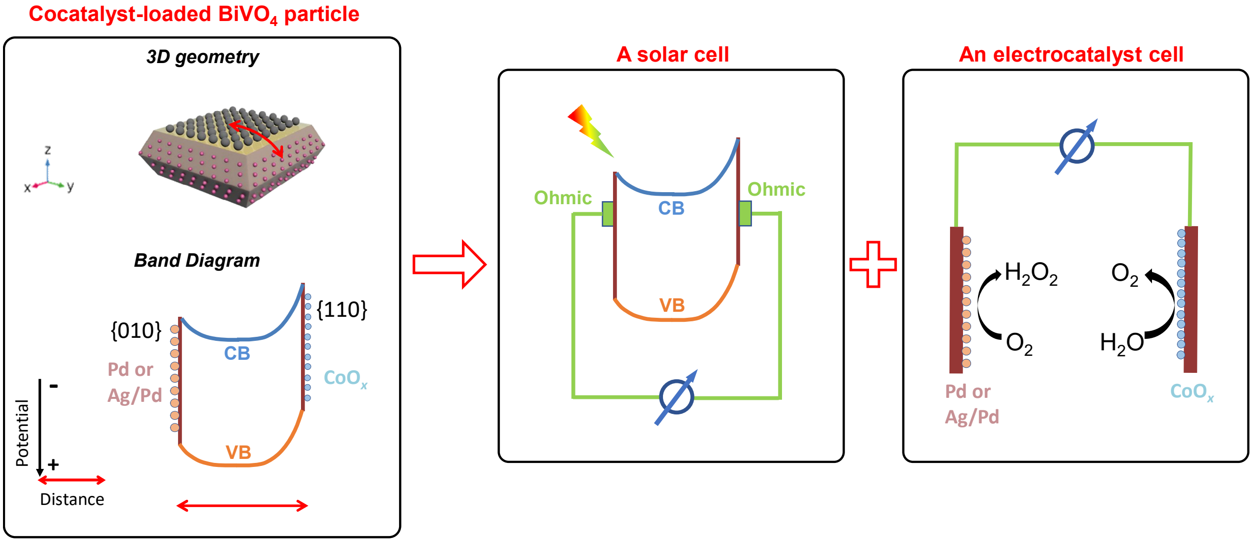

Figure S20. Simulation model for photocatalytic H_2O_2 generation by cocatalyst-loaded $BiVO_4$.

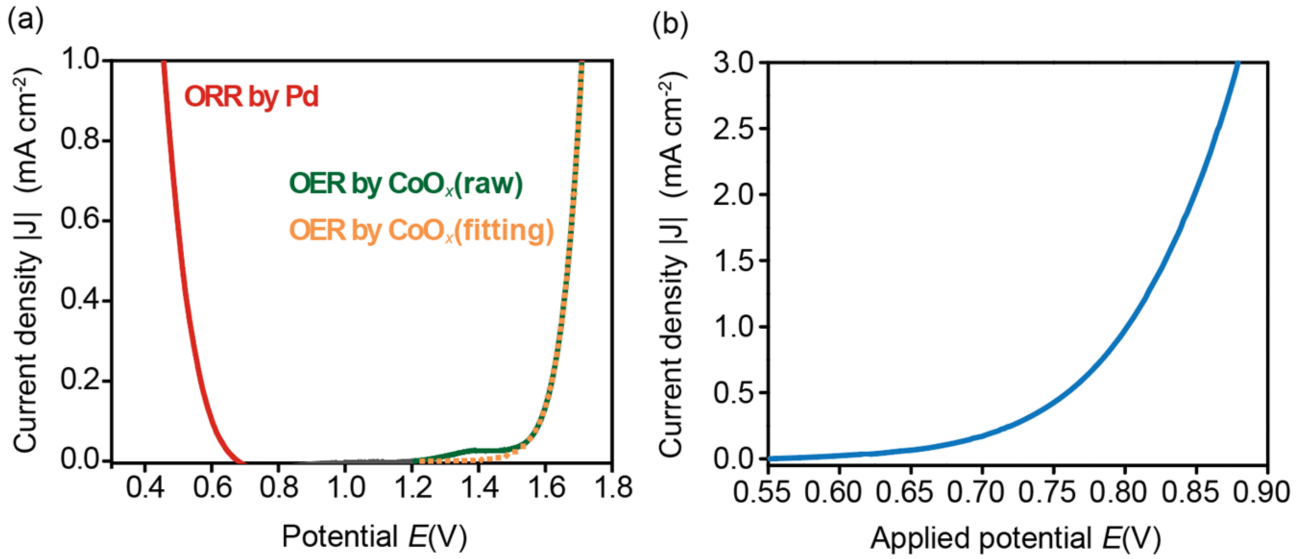

Figure S21. (a) *J-V* curves for O_2 evolution reaction (OER) on the anode with CoO_x and H_2O_2 generation (O_2 reduction reaction, ORR) on the cathode with Pd. The electrolyte is 0.5 M Na_2SO_4(aq) solution purged with O_2 without pH adjustment. A rotating disk electrode at 6000 rpm is used as a working electrode. (b) *J-V* curve for the electrocatalyst cell composed of a cathode with Pd for H_2O_2 generation and an anode with CoO_x for O_2 evolution.

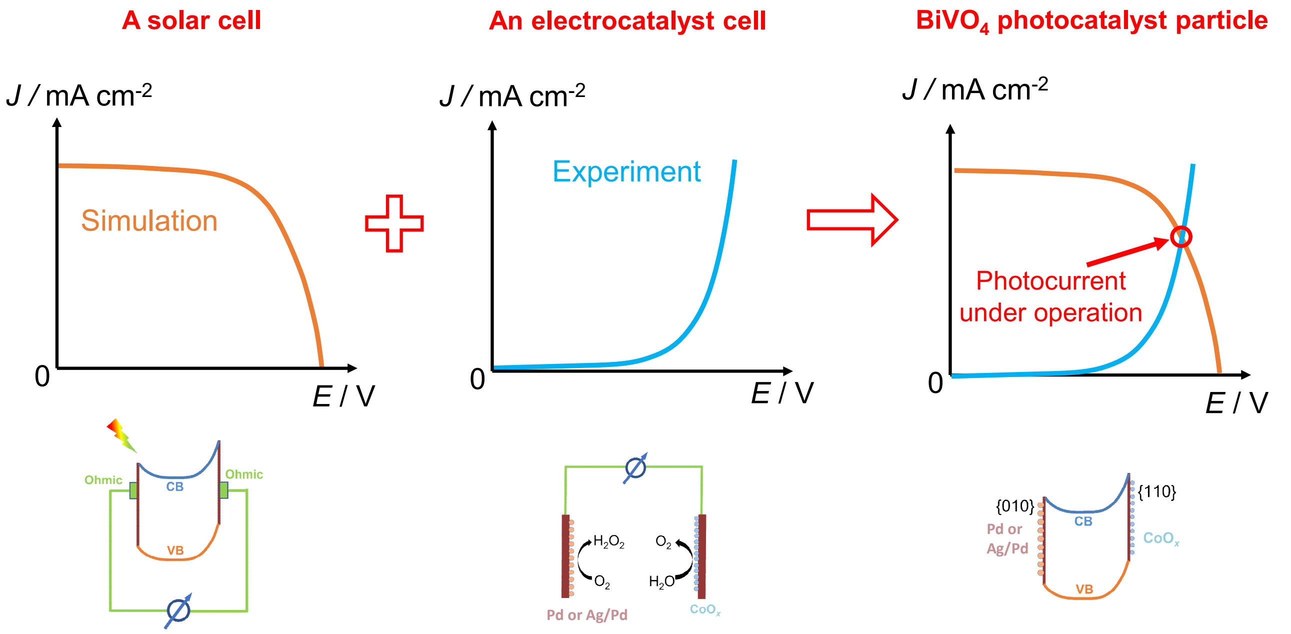

Figure S22. Schematic of combining the *J-V* curves of the solar cell and the electrocatalyst cell to obtain the photocurrent density of cocatalyst-loaded $BiVO_4$.

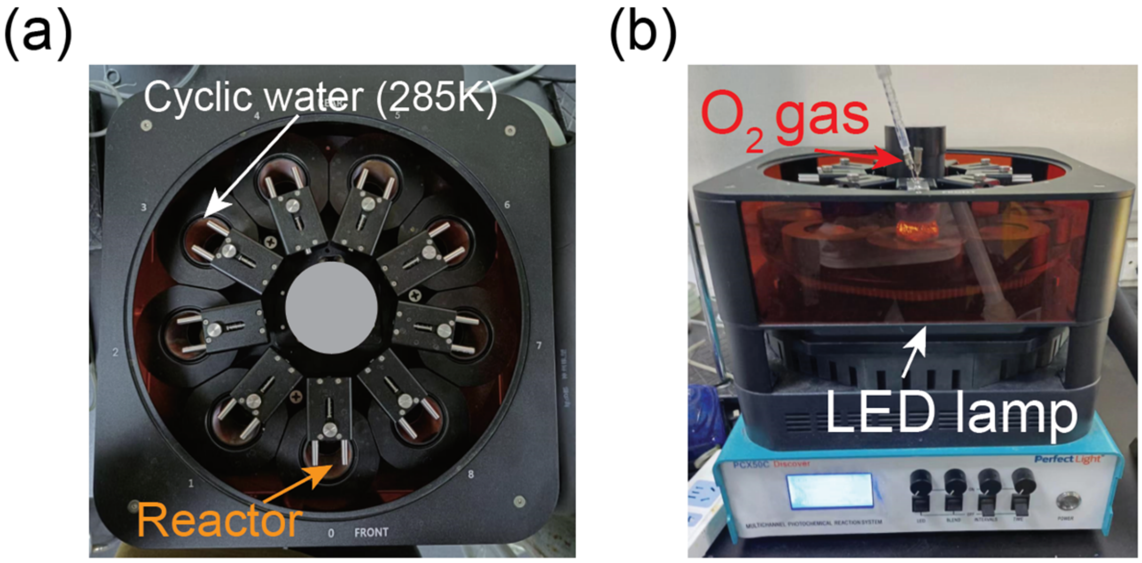

Figure S23. LED photosynthesis system used in the photocatalysis performance tests. (a) top view; (b) side view.

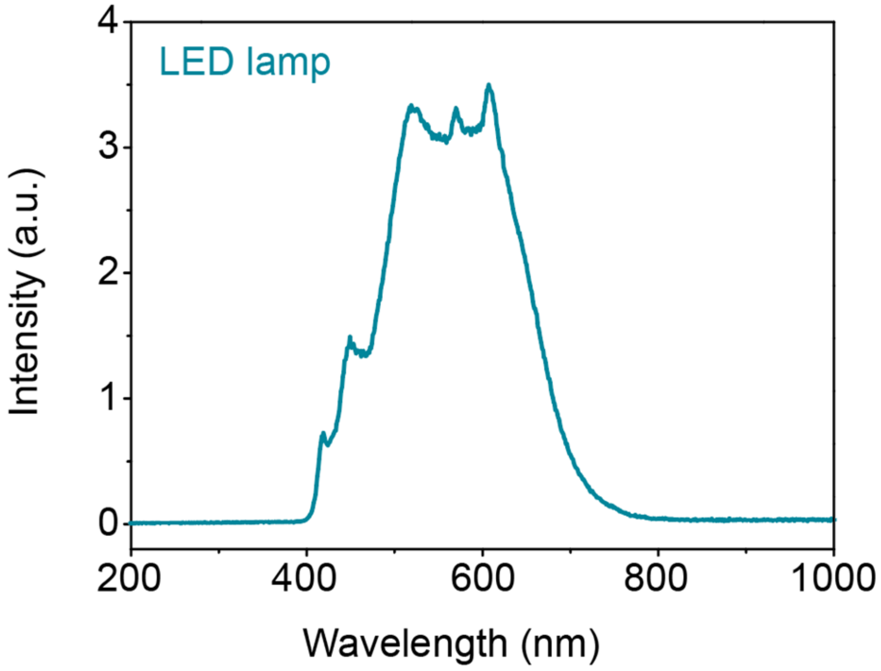

Figure S24. Spectrum of the LED lamp.

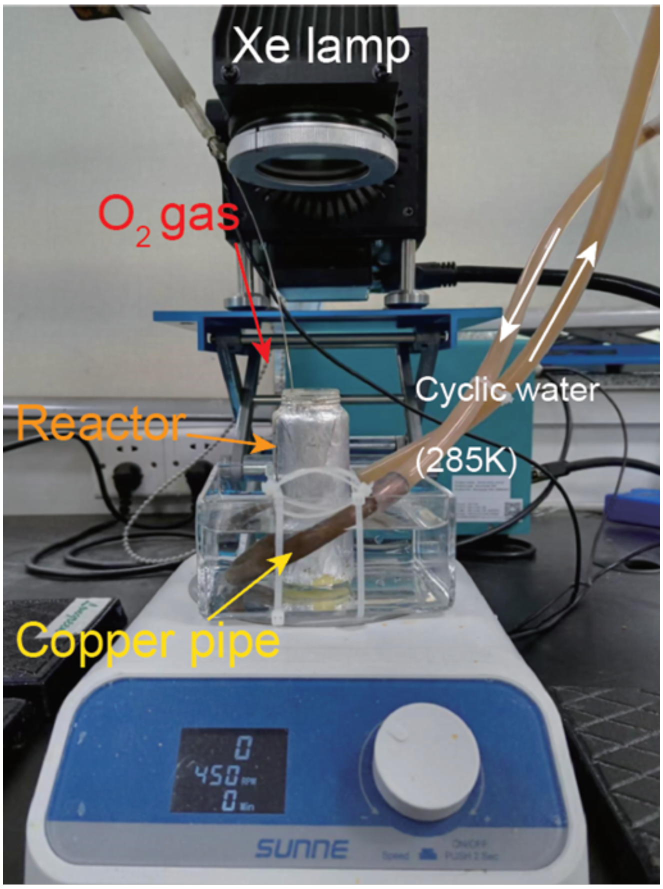

Figure S25. Xe lamp and reactor used in the photocatalysis performance tests.

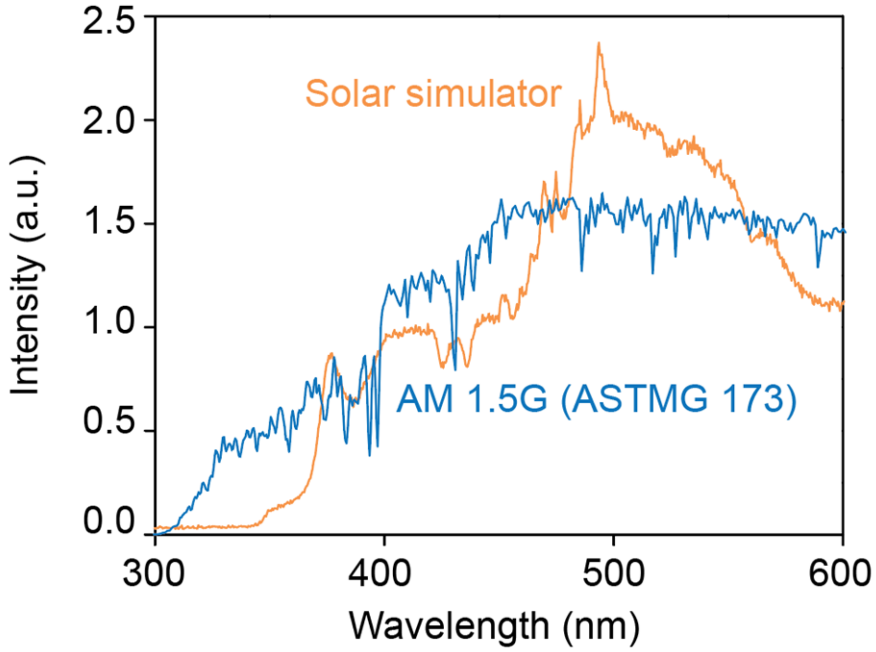

Figure S26. Spectrum of the Xenon lamp (orange line) and the standard AM 1.5G (blue line, ASTMG 173).

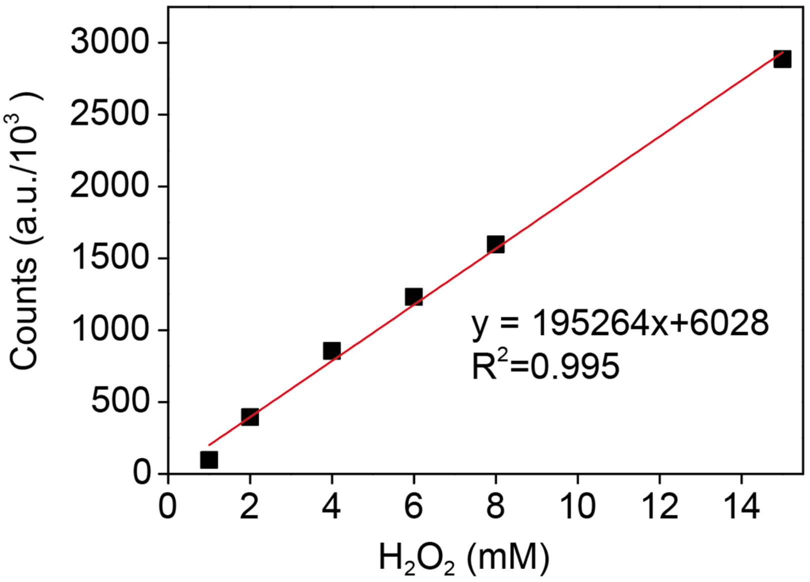

Figure S27. Calibration curve for quantifying photogenerated H_2O_2. The H_2O_2 concentration was calculated following the equation: Counts = 195264[H_2O_2] + 6028. For example, HPLC analysis of 60-min H_2O_2 production by CoO_x/Mo:$BiVO_4$/(Ag/Pd) gave a signal of 1894067 (the signal of the sample after 160-fold diluted tested by HPLC is 5918, so totally the signal of 60-min H_2O_2 production is 160×5918=1894067), corresponding to a H_2O_2 concentration of 9.7 mM.

Supplementary References

1 Liu, T. *et al.* Overall photosynthesis of H_2O_2 by an inorganic semiconductor. *Nat. Commun.* **13**, 1034 (2022).

2 Chu, C. H. *et al.* Electronic tuning of metal nanoparticles for highly efficient photocatalytic hydrogen peroxide production. *ACS Catal.* **9**, 626-631 (2019).

3 Moon, G. H., Kim, W., Bokare, A. D., Sung, N. E. & Choi, W. Solar production of H_2O_2 on reduced graphene oxide-TiO_2 hybrid photocatalysts consisting of earth-abundant elements only. *Energ Environ. Sci.* **7**, 4023-4028 (2014).

4 Hou, W. C. & Wang, Y. S. Photocatalytic generation of H2O2 by graphene oxide in organic electron donor-free condition under sunlight. *ACS Sustain. Chem. Eng.* **5**, 2994-3001 (2017).

5 Hirakawa, H. *et al.* Au nanoparticles supported on $BiVO_4$: effective inorganic photocatalysts for H_2O_2 production from water and O2 under visible light. *Acs Catal.* **6**, 4976-4982 (2016).

6 Shiraishi, Y., Matsumoto, M., Ichikawa, S., Tanaka, S. & Hirai, T. Polythiophene-doped resorcinol-formaldehyde resin photocatalysts for solar-to-hydrogen peroxide energy conversion. *J. Am. Chem. Soc.* **143**, 12590-12599 (2021).

7 Kou, M. P. *et al.* Molecularly engineered covalent organic frameworks for hydrogen peroxide photosynthesis. *Angew. Chem. Int. Edit.* **61**, e202200413 (2022).

8 Shiraishi, Y. *et al.* Resorcinol-formaldehyde resins as metal-free semiconductor photocatalysts for solar-to-hydrogen peroxide energy conversion. *Nat. Mater.* **18**, 985-995 (2019).

9 Teng, Z. Y. *et al.* Atomically dispersed antimony on carbon nitride for the artificial photosynthesis of hydrogen peroxide. *Nat. Catal.* **4**, 374-384 (2021).

10 Chu, C. H. *et al.* Spatially separating redox centers on 2D carbon nitride with cobalt single atom for photocatalytic H2O2 production. *Proc. Natl. Acad. Sci. U.S.A.* **117**, 6376-6382 (2020).

11 Kofuji, Y. *et al.* Graphitic carbon nitride doped with biphenyl diimide: efficient photocatalyst for hydrogen peroxide production from water and molecular oxygen by sunlight. *ACS Catal.* **6**, 7021-7029 (2016).

12 Kofuji, Y. *et al.* Carbon nitride-aromatic diimide-graphene nanohybrids: metal-free photocatalysts for solar-to-hydrogen peroxide energy conversion with 0.2% efficiency. *J. Am. Chem. Soc.* **138**, 10019-10025 (2016).

13 Zhu, Z. D., Pan, H. H., Murugananthan, M., Gong, J. Y. & Zhang, Y. R. Visible light-driven photocatalytically active g-C_3N_4 material for enhanced generation of H_2O_2. *Appl. Catal. B-Environ.* **232**, 19-25 (2018).

14 Ye, Y. X. *et al.* Highly efficient photosynthesis of hydrogen peroxide in ambient conditions. *Proc. Natl. Acad. Sci. U.S.A.* **118** (2021).

15 Tian, Q. *et al.* Nanospatial charge modulation of monodispersed polymeric microsphere photocatalysts for exceptional hydrogen peroxide production. *Small* **17**, 2103224 (2021).

16 Chen, X. L., Kuwahara, Y., Mori, K., Louis, C. & Yamashita, H. A hydrophobic titanium doped zirconium-based metal organic framework for photocatalytic hydrogen peroxide production in a two-phase system. *J. Mater. Chem. A* **8**, 1904-1910 (2020).

17 Chen, L. *et al.* Simultaneously tuning band structure and oxygen reduction pathway toward high-efficient photocatalytic hydrogen peroxide production using cyano-rich graphitic carbon nitride. *Adv. Funct. Mater.* **31**, 2105731 (2021).

18 Zhao, Z. Y., Li, Z. S. & Zou, Z. G. Electronic structure and optical properties of monoclinic clinobisvanite $BiVO_4$. *Phys. Chem. Chem. Phys.* **13**, 4746-4753 (2011).

19 Ma, Y. M., Pendlebury, S. R., Reynal, A., Le Formal, F. & Durrant, J. R. Dynamics of photogenerated holes in undoped BiVO4 photoanodes for solar water oxidation. *Chem. Sci.* **5**, 2964-2973 (2014).

20 Abdi, F. F., Savenije, T. J., May, M. M., Dam, B. & van de Krol, R. The origin of slow carrier transport in $BiVO_4$ thin film photoanodes: a time-resolved microwave conductivity study. *J. Phys. Chem. Lett.* **4**, 2752-2757 (2013).

21 Kawaguchi, K., Chugenji, T., Okunaka, S., Tokudome, H. & Katayama, K. Distinction and separation of different types of charge carriers from the time-resolved local charge carrier mapping for photocatalytic materials. *J. Phys. Chem. C* **126**, 6646-6652 (2022).

22 Takanabe, K. Photocatalytic water splitting: quantitative approaches toward photocatalyst by design. *ACS Catal.* **7**, 8006-8022 (2017).
